# Development of an AAV9-RNAi-mediated silencing strategy to abrogate TRPM4 expression in the adult heart

**DOI:** 10.1007/s00424-021-02521-6

**Published:** 2021-02-13

**Authors:** Rebekka Medert, Andreas Jungmann, Staffan Hildebrand, Martin Busch, Dirk Grimm, Veit Flockerzi, Oliver J. Müller, Patrick Most, Dagmar Schumacher, Marc Freichel

**Affiliations:** 1 Institute of Pharmacology, Heidelberg University, Heidelberg, Germany; 2 DZHK (German Centre for Cardiovascular Research), partner site Heidelberg/Mannheim, Heidelberg, Germany; 3 Department of Internal Medicine III, Cardiology, University Hospital Heidelberg, Heidelberg, Germany; 4 Institute of Pharmacology and Toxicology, University Clinic of Bonn, Bonn, Germany; 5 Department of Infectious Diseases/Virology, BioQuant, Medical faculty, Heidelberg University, Heidelberg, Germany; 6 DZIF (German Centre for Infection Research), partner site Heidelberg, Berlin, Germany; 7 Institute of Experimental and Clinical Pharmacology and Toxicology, Center for Molecular Signaling (PZMS), Saarland University, Homburg, Germany; 8 Department of Internal Medicine III, University of Kiel, Kiel, Germany; 9 DZHK (German Centre for Cardiovascular Research), partner site Hamburg/Kiel/Lübeck, Kiel, Germany

**Keywords:** Transient receptor potential (TRP) channel, TRPM4, Adeno-associated virus serotype 9 (AAV9), RNAi, Gene therapy

## Abstract

The cation channel transient receptor potential melastatin 4 (TRPM4) is a calcium-activated non-selective cation channel and acts in cardiomyocytes as a negative modulator of the L-type Ca^2+^ influx. Global deletion of TRPM4 in the mouse led to increased cardiac contractility under β-adrenergic stimulation. Consequently, cardiomyocyte-specific inactivation of the TRPM4 function appears to be a promising strategy to improve cardiac contractility in heart failure patients. The aim of this study was to develop a gene therapy approach in mice that specifically silences the expression of TRPM4 in cardiomyocytes. First, short hairpin RNA^miR30^ (shRNA^miR30^) sequences against the TRPM4 mRNA were screened in vitro using lentiviral transduction for a stable expression of the shRNA cassettes. Western blot analysis identified three efficient shRNA^miR30^ sequences out of six, which reduced the endogenous TRPM4 protein level by up to 90 ± 6%. Subsequently, the most efficient shRNA^miR30^ sequences were delivered into cardiomyocytes of adult mice using adeno-associated virus serotype 9 (AAV9)-mediated gene transfer. Initially, the AAV9 vector particles were administered via the lateral tail vein, which resulted in a downregulation of TRPM4 by 46 ± 2%. Next, various optimization steps were carried out to improve knockdown efficiency in vivo. First, the design of the expression cassette was streamlined for integration in a self-complementary AAV vector backbone for a faster expression. Compared to the application via the lateral tail vein, intravenous application via the retro-orbital sinus has the advantage that the vector solution reaches the heart directly and in a high concentration, and eventually a TRPM4 knockdown efficiency of 90 ± 7% in the heart was accomplished by this approach. By optimization of the shRNA^miR30^ constructs and expression cassette as well as the route of AAV9 vector application, a 90% reduction of TRPM4 expression was achieved in the adult mouse heart. In the future, AAV9-RNAi-mediated inactivation of TRPM4 could be a promising strategy to increase cardiac contractility in preclinical animal models of acute and chronic forms of cardiac contractile failure.

**Supplementary Information:**

The online version contains supplementary material available at 10.1007/s00424-021-02521-6.

## Introduction

TRPM4 is a Ca^2+^-activated, non-selective cation channel whose expression has been detected in the human heart as well as in the atrium and ventricle of the mouse [18, 22]. In humans, gain-of-function mutations of the *Trpm4* gene are strongly associated with familial arrhythmia syndromes such as Brugada syndrome and progressive familial heart block type I [17, 20, 35]. Previous studies showed that TRPM4-deficient mice have an increased β-adrenergic response compared to wild-type mice [23]. In pressure-volume (PV-loop) measurements, β-adrenergic stimulation with isoproterenol (ISO) resulted in an increased left ventricular contractility compared to control animals. In vitro studies on isolated cardiomyocytes indicated that TRPM4 deficiency is associated with a faster repolarization of the action potential and an increased Ca^2+^ influx via L-type channels with isoproterenol stimulation [23]. Recently, we could show that the increased ISO-evoked inotropy and elevated Ca^2+^ transient can only be observed in TRPM4-deficeint mice on the 129SvJ genetic background and the TRPM4 protein expression level is about 80% higher in wild-type 129SvJ mice than in mice with C57B/6N background [26]. The results of this study indicate that the relevance of TRPM4 for cardiac contractility depends on homeostatic TRPM4 expression levels or the genetic background of different mouse strains. Studies of cardiac contractility on isolated left ventricular papillary muscles also showed an increased contractile force in the papillary muscle of TRPM4^-/-^ mice compared to wild-type controls under β-adrenergic stimulation [37]. In the same study, it was additionally observed that the activation of adenylate cyclase in the papillary muscle of the TRPM4^-/-^ mouse resulted in a stronger contraction force than in papillary muscles of wild-type controls. In summary, both studies suggest that TRPM4 reduces the driving force for Ca^2+^ to enter cells via the L-type Ca^2+^ channels [23, 37]. From a translational point of view, inactivating cardiac TRPM4 function in cardiomyocytes is a promising approach to increase the ventricular contractility of the heart. Blocking cardiac TRPM4 function, especially at elevated catecholamine levels, as they occur in patients with chronic heart failure, could lead to an improvement in cardiac function. So far, there are no small compounds blocking TRPM4 channels with sufficient specificity. However, if such TRPM4 blockers existed, adverse effects could be expected from ubiquitous TRPM4 inactivation arising from other TRPM4-expressing cell types. Blocking the TRPM4 channel in chromaffin cells of the adrenal medulla could lead to hypertension, in mast cells to increased release of pro-inflammatory substances, and in immune cells to increased activation [22, 38]. Therefore, molecular inhibition of TRPM4 biosynthesis using RNA interference (RNAi) in combination with viral vector delivery to defined cell types is a suitable alternative to modify TRPM4 function in selected organ functions. Today, targeted RNAi strategies not only are used in basic research to downregulate gene expression, but also are becoming increasingly important for therapeutic use to inhibit expression of pathological relevant genes by harnessing the endogenous RNAi processing pathway [1, 13]. Classical short hairpin RNAs (shRNAs) under the control of strong RNA Pol III promoters can lead to saturation of the endogenous miRNA processing pathway, resulting in downregulation of endogenous miRNAs that fulfill vital functions [9, 11]. shRNAs embedded in an miRNA background (shRNA^miR^) enable the shRNA sequence to be transcribed efficiently by a tissue-specific RNA Pol II promoter. In contrast to Pol III promoter-driven shRNAs, less toxic effects were observed when using shRNA^miR^ constructs [5, 24]. This may be due to weaker RNA Pol II promoters, which lead to a lower expression level of the shRNA^miR^ cassette. Overall, the artificial shRNA^miR^ has an improved safety profile compared to classic shRNA, which is particularly important for gene therapy approaches. Because of the short DNA sequence and the simple design, shRNA and shRNA^miR^ expression cassettes can be packaged in AAV vectors to ensure stable and long-term expression in non-dividing cells [10]. For targeting of the myocardium in rodents, the AAV serotype 9 is particularly well suited after intravenous administration via the tail vein, evidenced by the finding that an AAV9 dose of 10^11^ vector genome copies per mouse typically results in robust transduction of over 90% of the cardiomyocytes in the mouse heart [14, 34]. However, it should be noted that AAV9 transduction is not limited to cardiomyocytes, and that other organs including the liver and brain can also be transduced to a high extent [43]. By using a cardiomyocyte-specific promoter, expression can be further restricted to cardiomyocytes [27, 32, 34, 41].

The aim of our work was to develop a highly efficient therapeutic AAV9-RNAi strategy to abrogate TRPM4 expression in the adult heart.

## Methods

### Plasmid construction

Four commercially available shTRPM4^miR30^ (#1-#4) constructs from Thermo Scientific (USA) were selected and delivered in a lentiviral pGIPZ vector. shTRPM4^mir-30^ sequences (Table S1) were flanked by recognition sites for the restriction enzymes XhoI (5′) and MluI (3′), to facilitate an exchange of the specific stem sequences. Two additional shTRPM4^mir-30^ (#5-# 6) constructs were generated. Their sequences were derived from commercially available shRNAs (shRNA-#5 - TRCN0000068684, shRNA-#6 - TRCN0000068687, Sigma, USA), and the sequence of shRNA #5 has already been shown to mediate robust downregulation of TRPM4 in mouse cells [29, 30]. The stem sequences of the two shRNAs were synthesized and cloned into the pGIPZ vector via a directed ligation using the flanking restriction enzyme recognition sites XhoI (5′) and MluI (3′). In a negative control vector, the shRNA^miR30^ sequence was removed using XhoI and MluI.

The shTRPM4^miR30^-tandem cassettes, containing shTRPM4^miR30^-#3 and shTRPM4^miR30^-#4, were synthesized with the restriction site XhoI (5′ direction) and SalI (3′ direction). The eGFP cDNA was PCR amplified containing the restriction sites XhoI (5′) and SalI (3′). Next, the shTRPM4^miR30^-tandem cassettes of the PCR-amplified eGFP were subcloned an XhoI-cut and SalI-cut scAAV recipient plasmid [4]. In addition, the expression cassettes comprising three different classic shRNAs against TRPM4 (shTRPM4-#7, shTRPM4-#8, and shTRPM4-#4) under the control of three different Pol III promoters were synthesized. shTRPM4#-4 has the same sense sequence as shTRPM4^mir30^-#4. The other two shRNAs (shTRPM4-#7, shTRPM4-#8) were generated via the online programs siRNA Wizard (https://www.invivogen.com/sirnawizard/) and Block-it RNAi Designer (https://rnaidesigner.thermofisher.com/rnaiexpress/rnaiDesign.jsp). Again, the expression cassette including the restriction sites XhoI (5′ direction) and SalI (3′ direction) was synthesized and subcloned into the EcoRI-cut and SalI-cut scAAV backbone.

### Generation and quantification of AAV9 viral particles

Recombinant AAV9 (rAAV9) vector particles were generated and purified using the iodixanol gradient ultracentrifugation method [8, 44]. The rAAV9 production was carried out using HEK293T cells. First, 1.8 × 10^8^ HEK293T cells were seeded in a ten chamber CellStack® (Corning, USA) and cultured in DMEM+Glutamax (Gibco, Thermo Fisher Scientific, USA) supplemented with 10% fetal bovine serum (FBS) and 1% Pen/Strep. After 48 h, a 1:1 molar ratio of rAAV vector plasmid and the *rep*-*cap* AAV9 helper plasmid (pDP9rs) was mixed and transfected using polyethyleneimine (PEI). The cells were harvested in 3 ml lysis buffer and lysed by four freeze-thaw cycles 72 h after transfection. The vector particles were purified using an iodixanol gradient consisting of four phases with decreasing density (60%, 40%, 25%, 15%) and ultracentrifugation at 50.000*g* for 135 min at 4 °C. Approximately 3 ml of the 40% phase, in which predominantly full virus particles accumulate, was recovered with a 27G needle. Finally, the vector solution was buffered into PBS using dialysis tubes (Zeba Spin Desalting Columns 7K MWCO, Thermo Scientific, USA) and concentrated (VivaSpin 10K MWCO, Sartorius, Germany). Vector titer was determined by quantifying vector genomes using a qPCR SYBR-Green assay (Biorad) and primers listed in Table S2 [16].

### Mouse experiments and genotyping

Experimental procedures were approved by the Regierungspräsidium Karlsruhe according to the Tierschutzgesetz (T-64/18, G-89/15). TRPM4-deficient mouse lines were described previously [38] and housed at the Interfaculty Biomedical Faculty (IBF) Heidelberg. Mice were kept under specified pathogen-free conditions on a 12-h light/12-h dark cycle with water and standard food (Rod18, LASvendi GmbH, Germany) available ad libitum. Injections of AAV9 at 3 × 10^12^ vector genomes per mouse (vg/mouse) were performed in 3-week to 8-week-old male mice. Tail vein (TV) injections were performed in conscious-restrained mice by injecting 200 μl vector solution into the lateral vein. Retro-orbital sinus injections of 100 μl vector solution were performed in anesthetized mice using 2% isoflurane. Genotyping of mice carrying a *trpm4*^*−*^ (null) allele, a *trpm4*^*flox*^ allele [38], and the a-MHC-Cre^ERT2^ transgene [36] and their wild-type siblings was carried out by PCR using the primer pairs listed in Table S2. To obtain template genomic (g)DNA, ear biopsies were taken and transferred into 97.5 μl of DirectPCR buffer (Viagen, USA) supplemented with 2.5 μl proteinase K (10 mg/ml, AppliChem, Germany) and lysed overnight at 55 °C. Proteinase K was denatured for 45 min at 85 °C. Taq PCR (0.3 μl Taq DNA Polymerase (lab made), 1x reaction buffer (100 mM Tris pH 8.3, 15 mM MgCl_2_, 500 mM KCl), 200 μM dNTPs, 500 nM primer forward and reverse and 1.0 μl gDNA) was performed using the following PCR protocol: 94 °C 30 s denaturation, 35 cycles of denaturation at 94 °C for 10 s, annealing at 58 °C for 30 s, and elongation at 72 °C for 30 s.

### Microsomal membrane preparation

Mice were sacrificed using cervical dislocation. The thorax was opened and the still beating heart was washed out with 0.9% NaCl (Braun, Germany) injected via the apex. Mouse hearts were transferred in PBS and atria were removed. Ventricular tissue was homogenized in 1 ml lysis buffer (100 mM TRIS-HCl, 1 mM MgCl_2_, pH 8.0 supplemented with 1 mM iodacetamide, 1 mM phenantholin, 0.1 mM PMSF, 1 μg/ml antipain, 1 μg/ml leupeptin, 0.7 μg/ml pepstatin, 1 mM benzamidine, 0.3 μM aprotinin, AppliChem, Germany) using a 2-ml tissue grinder (Tenbroeck Tissue Grinders, Wheaton, USA). Homogenates were filled up to 3 ml with lysis buffer and were frozen at −80 °C for 20 min following by thawing on ice for about 1 h. Afterwards, 15 ml of sucrose buffer (0.25 M sucrose; 10 mM TRIS-HCl; pH 7.4 supplemented with 1 mM iodacetamide, 1 mM phenantholin, 0.1 mM PMSF, 1 μg/ml antipain, 1 μg/ml leupeptin, 0.7 μg/ml pepstatin, 1 mM benzamidine, 0.3 μM aprotinin, Applichem, Germany) was added and the samples were centrifuged for 30 min at 6000*g*. Subsequently supernatant was centrifuged at 50.000*g* for 1 h and 45 min. The resulting microsomal membrane pellet was dissolved in 200-400 μl sucrose buffer. Protein concentration was determined by BCA assay (Thermo Scientific, USA). Microsomal membrane fractions were supplemented with 4x Laemmli buffer (60 mM TRIS-HCl, 10% (v/v) glycerol, 5% (v/v) β-mercaptoethanol, 4% (w/v) SDS, 0.005% (w/v) bromophenol blue; pH 6.8) and incubated at 60 °C for 20 min. Afterwards, microsomal membrane fractions were stored at −80 °C.

### Immunoblotting

Microsomal membrane fractions (50 μg/well) were loaded on a 4-12% Bis-Tris Plus or 10% Bis-Tris Plus gel (Invitrogen, USA) followed by gel electrophoresis at 130 V for 90 min. Resolved proteins were electroblotted onto a 0.45-μm protran nitrocellulose membrane (GE Healthcare, USA) at 12 mV for 60 min. Subsequently, the membrane was blocked in 5% non-fat milk with TBST (50 mM TRIS-HCl, 150 mM NaCl, 0.1% (v/v) Tween-20, pH 7.5) for 1 h. Membranes were incubated in anti-GAPDH (1:500 dilution, Acris, USA), anti-TRPM4 [38] (1:100, ab578), and anti-Calnexin (1:200, ab1039) antibodies at 4 °C for 24 h. Membranes were washed three times for 10 min in TBST and were then incubated in horseradish peroxidase-conjugated anti-rabbit antibody (1:50000, GE Healthcare, USA) at room temperature for 2 h. Membranes were washed two times for 10 min in TBST and once for 10 min in TBS (50 mM TRIS-HCl, 150 mM NaCl, pH 7.5). Afterwards, membranes were incubated in SignalFire Elite ECL (Cell Signaling, USA) for 1 min followed by the detection of chemiluminescence using digital imaging (GE Healthcare, ImageQuant LAS 4000 mini). Protein expression levels were measured by densitometry analysis using the ImageJ software.

### Droplet digital PCR

Droplet digital PCR (ddPCR) was carried out to determine the AAV genome copy number per host genome using the Magel2 gene as a diploid reference [28]. Primers and an FAM-labeled probe specific for the bGHpA (bovine growth hormone polyadenylation signal) sequence were used to determine the AAV genome copies (Table S2). Primers and an HEX-labeled probe were designed to determine the reference Magel2 gene copy number (Table S2).

Genomic DNA (gDNA) from the heart, liver, and skeletal muscle (musculus vastus lateralis) was isolated and purified by DNAeasy Blood and Tissue kit (Qiagen, USA), and then digested with HindIII (New England Biolabs, USA) for 1 h at 37 °C. ddPCR multiplex master mix was prepared by adding 11 μl 2 × Supermix for Probes (no UTPs) (Bio-Rad, USA), 1.1 μl 900 nM forward and reverse primers, 1.1 μl bGHpA Probe (250 nM), 1.1 μl Magel2 Probe (250 nM), 3.3 μl digested gDNA (7.5 ng/μl), and 3.3 μl ddH2O. Droplets were generated using a QX200 Droplet Generator (Bio-Rad, USA) according to the manufacturer’s protocol. The resulting droplets were transferred to a 96-well PCR plate (Thermos Scientific, USA) and end point PCR was performed using a C1000 Touch PCR thermal cycler (Bio-Rad) and the following PCR program: 95 °C for 5 min, 30 cycles of 95 °C for 30 s and 54 °C for 60 s, 4 °C for 5 min, 90 °C for 5 min, and then cooling down to 4 °C. Fluorescence FAM and HEX signals of end point PCR products were analyzed using the QX200 droplet reader (Bio-Rad, USA) and the gene copy numbers were analyzed using the QuantaSoft™ software (Bio-Rad, USA, version 1.7.4.0917). The AAV genome copy number per host genome was calculated as follows: bGHpA positive droplets/(agel2 positive droplets/2).

### Statistical analysis

Statistical analysis was performed using OriginPro 8.5 and Excel 2010. Data are expressed as mean ± standard deviation (SD). Statistical significance between the two groups was tested using a two-sample *t*-test: ***p < 0.0001, **p < 0.001, *p < 0.05.

## Results

### Maximum achievable myocardial TRPM4 protein knockdown: evaluation in Trpm4^fx/fx^/αMHC-Cre^ERT2^ mice

We reasoned that complete inactivation of the Trpm4 expression in cardiomyocytes via Cre-LoxP-mediated gene deletion represents the most useful indicator for a maximum achievable TRPM4 protein knockdown in ventricular tissue. Provided that a specific antibody is available, the easiest way to estimate the amount of protein present is by Western blotting. Our experience from analysis of other low abundant membrane proteins including TRPM4 tells that a membrane protein fraction must first be prepared from cells or tissues before running these proteins on SDS-PAGE and blotting them onto a membrane for reliable detection and quantification.

**Fig. 1:**
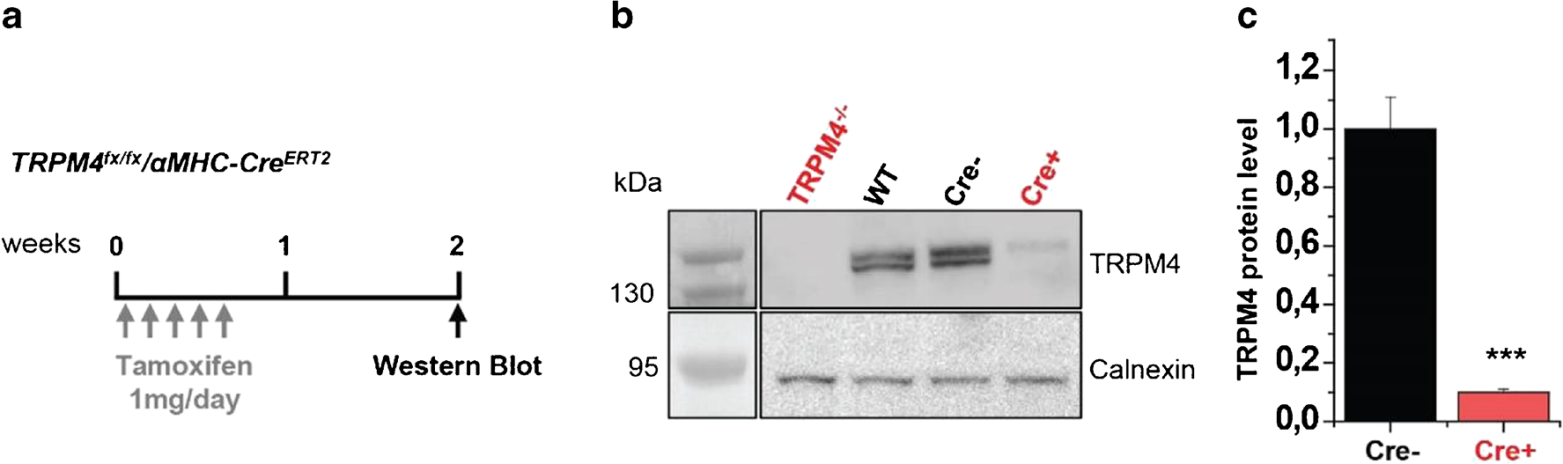
Evaluation of maximum achievable myocardial TRPM4 protein knockdown in cardiomyocyte-specific TRPM4 knockout mice. **a** Course of tamoxifen treatment in TRPM4^fx/fx^/αMHC-Cre^ERT2-postive^ and TRPM4^fx/fx^/αMHC-Cre^ERT2-Cre negative^ mice. **b** Western blot analysis with anti-TRPM4 and anti-Calnexin 2 weeks after tamoxifen injections. **c** Quantification of the relative TRPM4 protein level in the heart of TRPM4^fx/fx^/αMHC-Cre^ERT2-positive^ (Cre+) mice in comparison to TRPM4^fx/fx^/αMHC-Cre^ERT2-negative^ (Cre^−^) mice. Relative protein level of TRPM4 is expressed as mean ± SD. ***p <0.005

For the induction of the αMHC-Cre^ERT2^ recombinase, tamoxifen was administered intraperitoneally once a day on 5 consecutive days to 6-week-old Trpm4^flox/flox^/αMHC-Cre^ERT2-positive^ mice (Fig. 1a). Trpm4^flox/flox^/αMHC-Cre^ERT2-negative^ siblings served as controls and were also treated with tamoxifen. A reduction in TRPM4 protein levels was observed in ventricular microsomal membrane fractions of αMHC-Cre^ERT2-positive^ animals already 2 weeks after the last tamoxifen injection (Fig. 1b). Quantitative Western blot analysis in samples of three Cre-negative and six Cre-positive mice showed a TRPM4 reduction of 90 ± 1% in mice with induction of Cre expression in cardiomyocytes (Fig. 1c). Assuming efficient Cre-mediated recombination in cardiomyocytes, the remaining TRPM4 proteins must originate from cardiac cells other than cardiomyocytes, for example from cardiac fibroblasts and endothelial cells [36]. The result shown in Fig. 1b-c therefore suggests that a 90% reduction of TRPM4 is the maximum level that can be achieved by the intended knockdown approach.

### Evaluation of different shTRPM4^miR30^ sequences in vitro

**Fig. 2:**
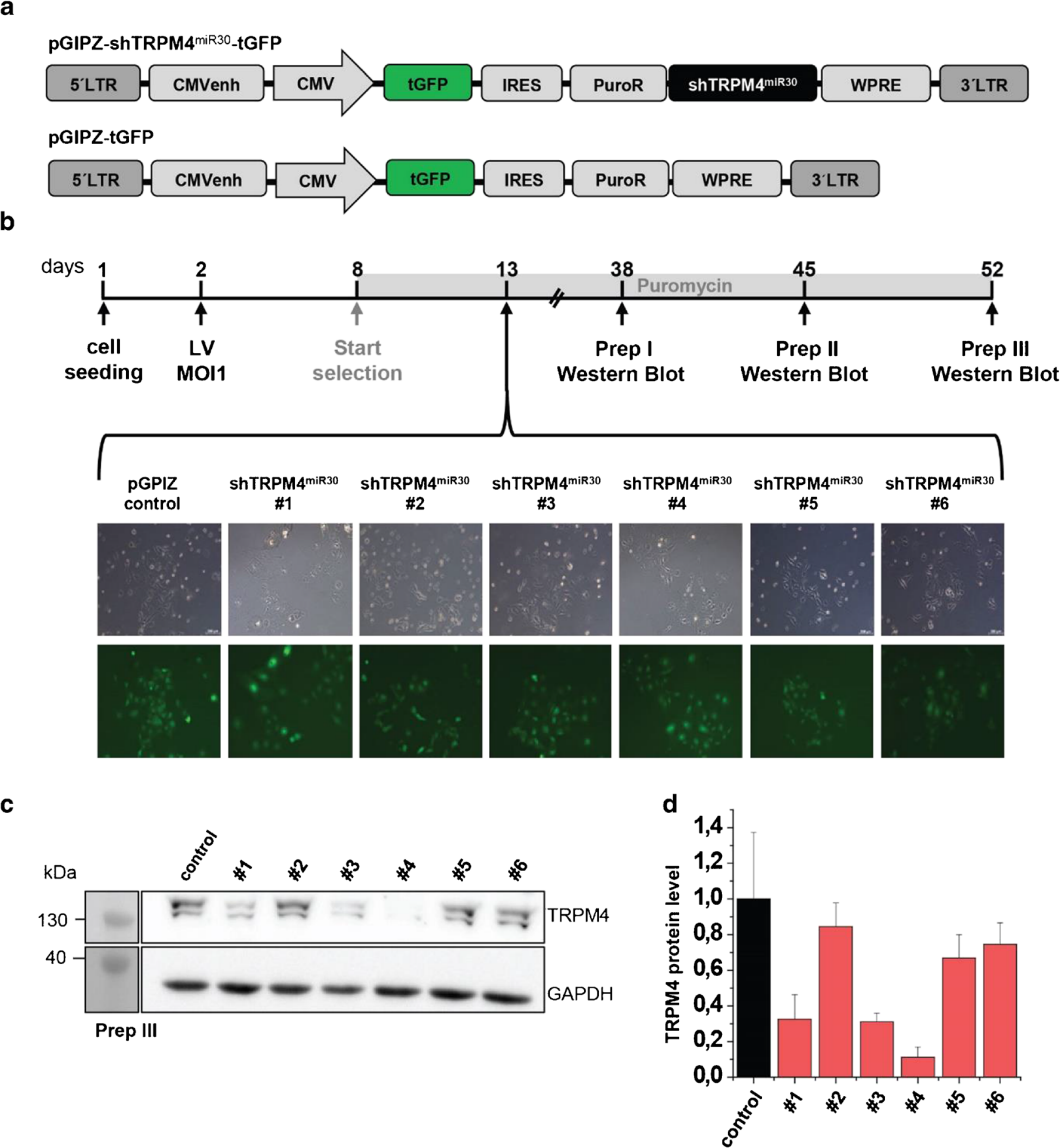
Evaluation of shTRPM4^miR30^ constructs in the murine B16F10 cell line. **a** Schematic representation of the lentiviral pGIPZ-shTRPM4^miR30^ vector and the pGIPZ control vector. **b** Experimental course of lentiviral transduction and analysis of the GFP fluorescence signal in puromycin-selected B16F10 cells. **c** Western blot analysis using microsomal membrane fractions of B16F10 cells expressing either of the six shTRPM4^miR30^(#1-#6) constructs to evaluate shRNA^miR30^-mediated TRPM4 knockdown. **d** Quantification of the relative TRPM4 protein expression level compared to pGIPZ-tGFP control-transduced B16F10 cells

To achieve a specific reduction of TRPM4 protein expression with the help of an RNAi strategy, four different commercially available microRNA-30-based shRNAs (shTRPM4^miR30^) targeting murine TRPM4 were chosen. The shTRPM4^miR30^ sequences were purchased from Thermo Scientific Open Biosystems in the lentiviral pGIPZ vector (Fig. 2a). In addition, two classic shRNA sequences (shRNA^miR30^ #5 and #6, Table S1) were tested based on previously published work [29, 30] and cloned into the lentiviral pGIPZ backbone. In order to evaluate their TRPM4 knockdown potential, individual shTRPM4^miR30^ sequences were subsequently tested in murine B16F10 cells. Screening of various cell lines regarding TRPM4 expression revealed that murine B16F10 cells do endogenously express TRPM4 proteins (Fig. 2c). All pGIPZ-shTRPM4^miR30^ constructs as well as a pGIPZ control construct, in which the shTRPM4^miR30^ cassette was removed, were packaged into lentiviral particles (Fig. 2a). The B16F10 cells were seeded at 100.000 cells per well and transduced 24 h later with a multiplicity of infection (MOI) of 1 (Fig. 2b). All pGIPZ-shTRPM4^miR30^ constructs contained a puromycin resistance cassette and selection was performed 6 days after transduction using puromycin (1 μg/ml). According to a FACS analysis, more than 99% of cells were GFP-positive on day 13 after selection (Fig. 2b and Fig. S1). Such cultures of B16F10 cells expressing the individual pGIPZ-shTRPM4^miR30^ constructs were expanded for Western blotting.

In total, the shTRPM4^miR30^-expressing B16F10 cells were expanded three times to obtain three independent replicates of microsomal membrane preparations and Western blot analysis (Fig. 2b-c and Fig. S2). Densitometric analysis of all three cell preparations showed that shTRPM4^miR30^-#4 generated the strongest TRPM4 reduction of 90 ± 6% in B16F10 cells compared to the pGIPZ-tGFP control, followed by shTRPM4^miR30^-#3 with an average TRPM4 reduction of 70 ± 5% and shTRPM4^miR30^-#1 with a reduction of 69 ± 14% (Fig. 2d).

### Myocardial TRPM4 knockdown obtained by a single-stranded AAV9-CMV-shTRPM4^miR30^-tandem construct

To use the shTRPM4^miR30^ constructs for TRPM4 knockdown in murine hearts, the constructs were transferred to an adeno-associated virus (AAV) vector system, and packaged as AAV serotype 9 that leads to highest transduction efficiency in cardiomyocytes when applied at doses of 1 × 10^11^-1.8 × 10^12^ vg/mouse via the lateral tail vein [14, 31, 34]. For the RNAi-based reduction of TRPM4, high cardiac transduction efficiency as well as a high AAV9 copy number per cardiomyocyte should be achieved by the application of 3 × 10^12^ vg/mouse.

**Fig. 3:**
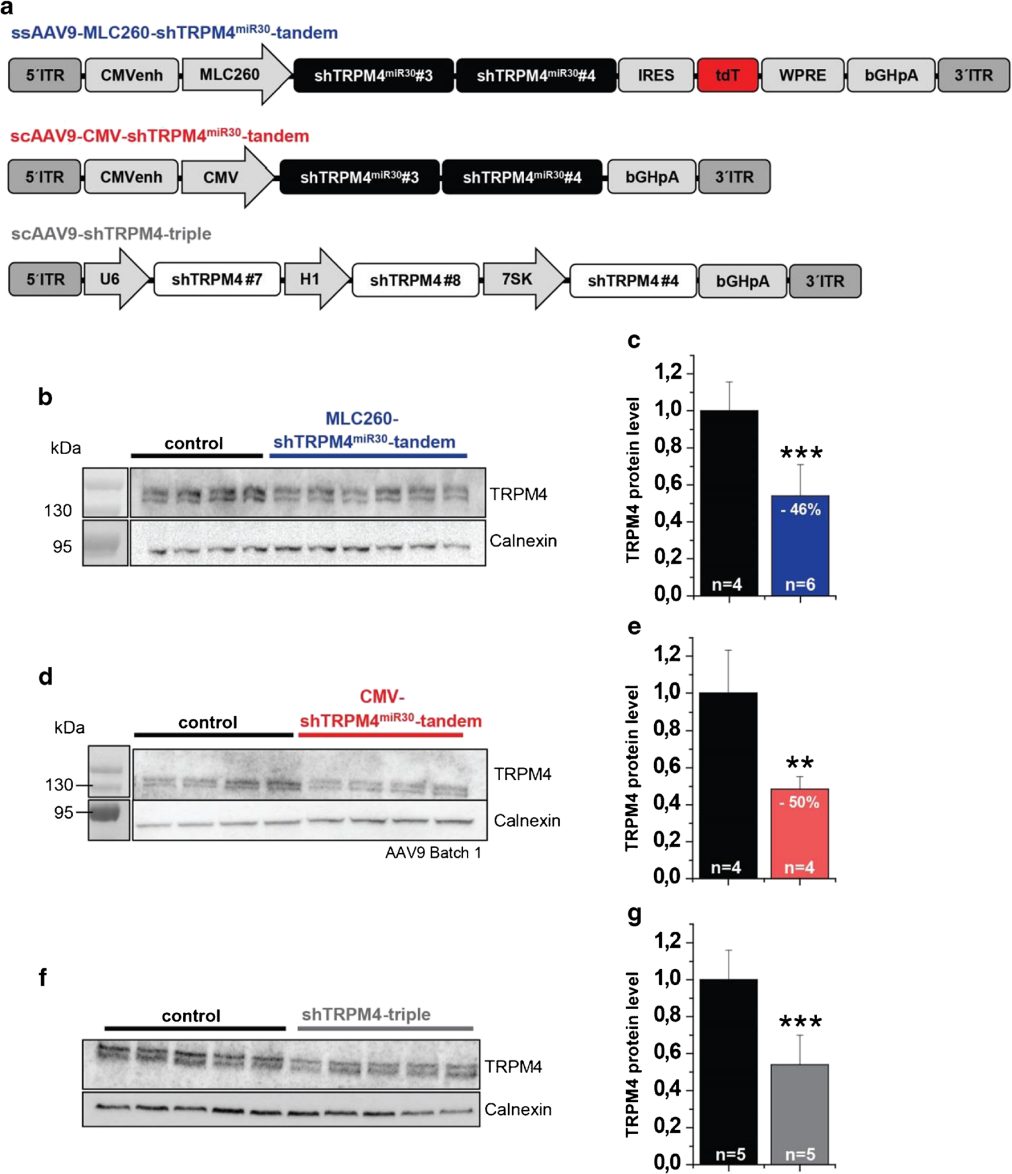
Evaluation of TRPM4 knockdown-mediated AAV9-RNAi constructs in the mouse heart 11 weeks after tail vein injection. **a** Schematic representation of AAV-RNAi vectors used. **b**-**g** Western blot analysis with anti-TRPM4 and anti-Calnexin antibodies in ventricular microsomal membrane fractions. Single lanes represent myocardial protein fractions of individual mice. **b**, **c** Analysis of TRPM4 expression in hearts of ssAAV9-MLC260-TRPM4^miR30^-tandem treated mice compared to untreated siblings. **d**, **e** Quantification of the relative TRPM4 protein level in TRPM4 protein expression in hearts of scAAV9-CMV-TRPM4^miR30^-tandem-treated mice. **f**, **g** Quantification of Western blot analysis in hearts of scAAV-shTRPM4-triple-treated mice. Relative expression of TRPM4 is expressed as mean ± SD. ***p <0.005

For the AAV9-mediated shTRPM4^miR30^ transfer into the mouse heart, an AAV vector was cloned, which contained the two most promising shTRPM4^miR30^ sequences (shTRPM4^miR30^-#3 and shTRPM4^mir30^-#4) as a tandem construct (termed “shTRPM4^miR30^-tandem”) under the control of the cardiomyocyte-specific MLC260 promoter (Fig. 3a, upper panel). Based on the size of the expression cassette and the packaging limit of AAV, a single-stranded AAV vector (ssAAV) had to be used.

Six 8-week-old male wild-type mice were treated with 3 × 10^12^ vg/mouse of the shTRPM4^miR30^-tandem construct. The vector was administered as single intravenous injection to the tail vein. As a control group, four untreated siblings were used, which were kept under the same conditions as the AAV9-treated mice. Western blot analysis of TRPM4 protein expression from microsomal membrane fractions of the myocardium was carried out 11 weeks after AAV9 application (Fig. 3b). Examination of the TRPM4 levels in the heart showed a significant reduction of 46 ± 17% in TRPM4 protein expression in the AAV9-shTRPM4^miR30^-tandem-treated mice compared to untreated siblings (Fig. 3b-c).

### Self-complementary scAAV9-CMV-shTRPM4^miR30^-tandem-mediated knockdown of TRPM4 in the myocardium of the mouse

Since the ssAAV-shTRPM4^miR30^-tandem construct did not achieve a sufficiently high knockdown efficiency, we considered to use a self-complementary AAV (scAAV) that is often more efficient [25]. Due to the limited packaging capacity of the scAAV genome (2.2 kb), the WPRE sequence, the IRES, and the reporter gene used in the previous construct were omitted. In addition, the cardiac MLC260 promoter was replaced by the enhanced human CMV promoter (Fig. 3a, middle panel), which is considered to convey higher expression levels in mouse cardiomyocytes [27]. In an alternative approach using the same vector backbone, an expression cassette consisting of three different classic shRNAs (shTRPM4-#7, shTRPM4-#8, and shTRPM4-#4) against TRPM4 under the control of three different RNA Pol III promoters was cloned (Fig. 3a, lower panel). In this construct, shTRPM4-#4 has the same sense sequence as in shTRPM4^mir30^-#4, which achieved 90% TRPM4 reduction in B16F10 when expressed after lentiviral transduction. The other two shRNAs (shTRPM4-#7, shTRPM4-#8) were generated via the online program siRNA Wizard and Block-it RNAi Designer.

The two experimental groups were treated with scAAV9-shTRPM4-triple and scAAV9-shTRPM4^miR30^-tandem vector particles, respectively. Four to five 8-week-old male mice were treated with 3 × 10^12^ vg/mouse, and four to five untreated siblings served as controls. Vectors were again applied by single intravenous injection via the tail vein. Analysis of TRPM4 protein expression was carried out 11 weeks after AAV9 application. Microsomal membrane proteins were prepared from hearts and the TRPM4 protein levels were analyzed by Western blotting. Densitometric evaluation of the antibody stain intensities showed a significant reduction in TRPM4 protein expression by 50 ± 7% in the heart of mice treated with the scAAV9-shTRPM4^mir30^-tandem vector (Fig. 3d-e) and by 46 ± 16% when treated with the scAAV9-shTRPM4-triple (Fig. 3f-g) vector compared to untreated controls.

**Fig. 4:**
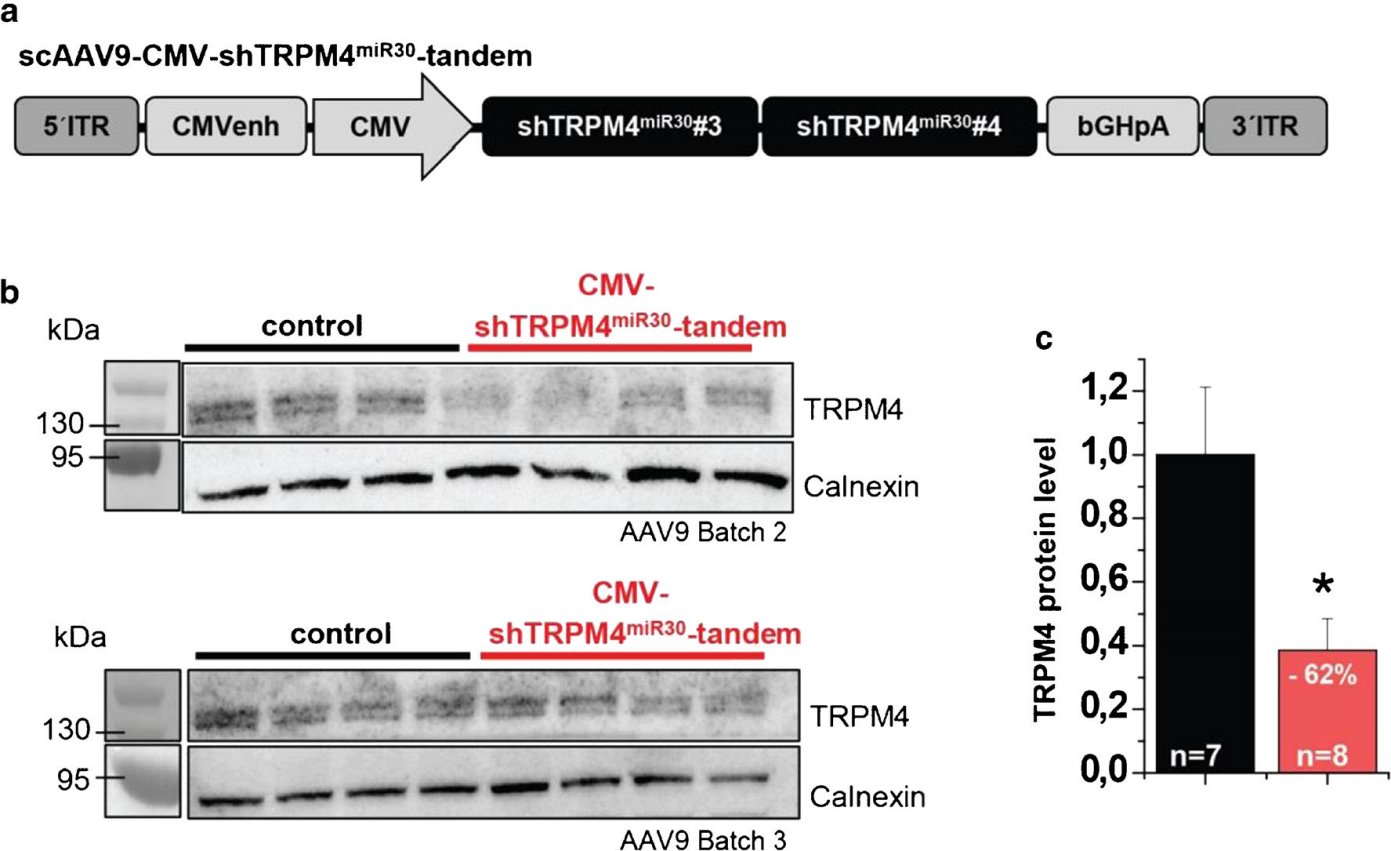
Evaluation of TRPM4 knockdown mediated by the scAAV9-CMV-TRPM4^miR30^-tandem vector in the mouse heart 16 weeks after tail vein injection. **a** Schematic representation of the vector used. **b** Western blot analysis with anit-TRPM4 and anti-Calnexin antibodies in ventricular microsomal membrane fractions after the application of AAV9 batch 2 (upper blot) or batch 3 (lower blot). Single lanes represent myocardial protein fractions of individual mice. **c** Quantification of the densitometric Western blot analysis of TRPM4 protein expression in hearts of scAAV9-CMV-TRPM4^miR30^-tandem treated mice compared to controls. Relative expression of TRPM4 is expressed as mean ± SD. *p<0.05, **p<0.01

To further study the kinetics and longevity of shTRPM4^miR30^-tandem expression and TRPM4 knockdown, animals treated with additional scAAV9-shTRPM4^mir30^-tandem production batch 2 and 3 were analyzed at a later time point, 16 weeks after AAV9 application (Fig. 4), using Western blotting (Fig. 4b) to evaluate whether the TRPM4 reduction would be even more pronounced after extended shRNA expression. Indeed, densitometric analysis 16 weeks after AAV application showed a downregulation of TRPM4 expression by 61 ± 9% in scAAV9-shTRPM4^mir30^-tandem treated mice compared to untreated siblings (Fig. 4c).

### Optimization of the scAAV9-CMV-shTRPM4^miR30^-tandem-mediated knockdown of TRPM4 in the myocardium of the mouse

**Fig. 5:**
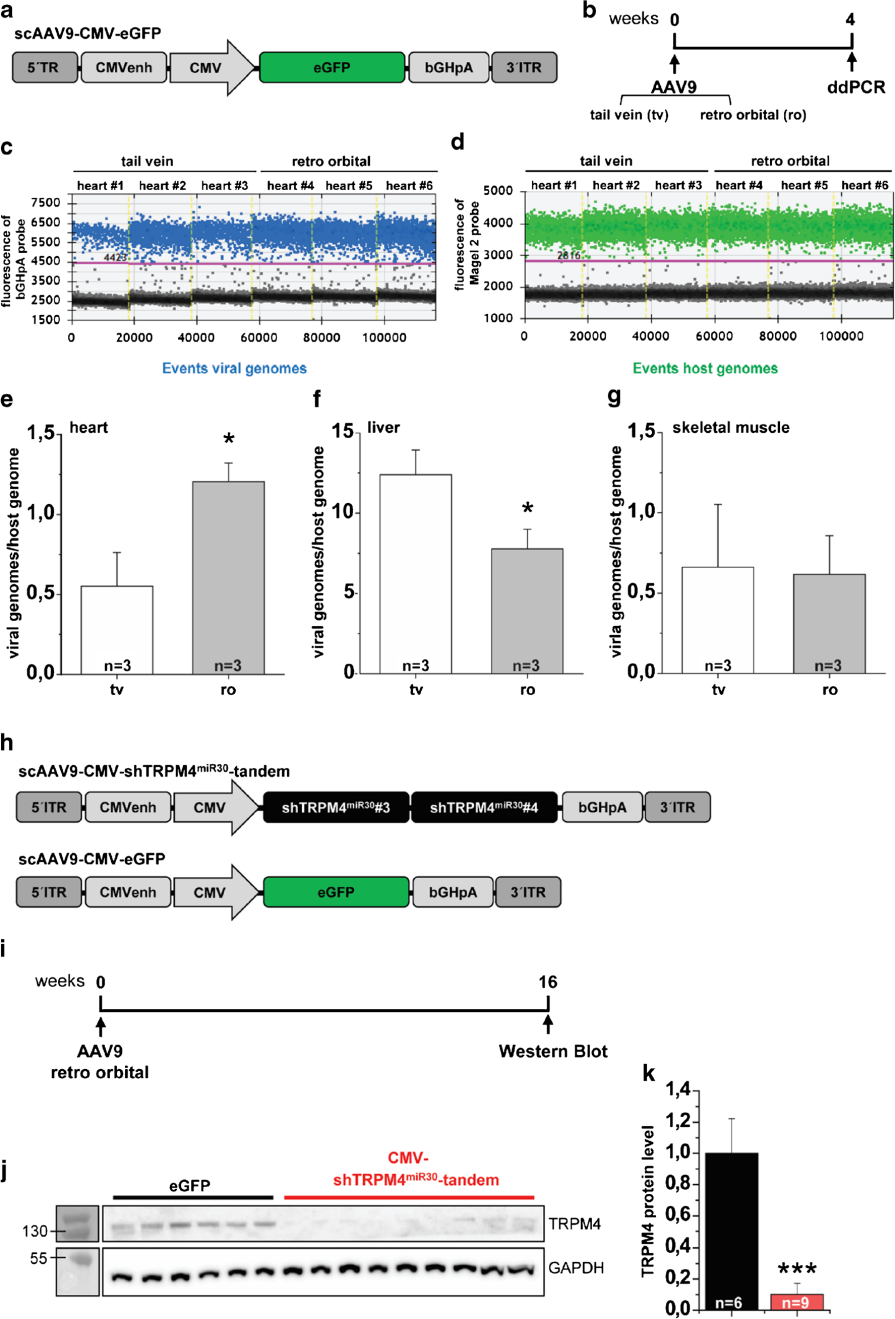
AAV9 application via the retro-orbital sinus elevates cardiac transduction efficiency and results in efficient scAAV9-CMV-shTRPM4^miR30^-tandem-mediated TRPM4 knockdown in the moue heart. **a** Comparison of the intravenous AAV9 application methods via the tail vein (sv) and the retro-orbital sinus (ro) of the mouse. **b** Schematic representation of the experimental course. **c-g** Determination of AAV9 transduction efficiency using droplet digital PCR (ddPCR). Droplet count view for **c** viral genomes and **d** host genomes in hearts injected via tail vein or retro-orbital sinus. Positive droplets are displayed in blue (viral genome counts of the AAV containing bGHpA, bovine growth hormone polyadenylation signal sequence) or green (host genome counts of the Magel2 reference gene) and negative droplets in black. AAV9 transduction efficiency was quantified as viral genomes/host genome in the **e** heart, **f** liver, and **g** skeletal muscle (musculus vastus lateralis). **h** Schematic representation of the scAAV-CMV-shTRPM4^miR30^-tandem and scAAV9-CMV-GFP control vector used. **i** Analysis of TRPM4 expression followed 16 weeks after AAV9 application. **j** Western blot analysis with anti-TRPM4 and anti-GAPDH antibodies of ventricular microsomal membrane fractions. Single lanes represent myocardial protein fractions of individual mice. **k** Quantification of the relative TRPM4 protein in hearts of scAAV9-CMV-TRPM4^miR30^-tandem-treated mice compared to AAV9-CMV-GFP treated controls. Relative expression of TRPM4 is expressed as mean ± SD. *p <0.05, ***p <0.005

Next, we studied the routes of AAV9 application and compared tail vein injection with AAV9 injection via the retro-orbital venous plexus. In a pilot study, mice in both experimental groups were treated with 1 × 10^11^ vg/mouse of a scAAV9-eGFP vector (Fig. 5a). Four weeks later, transduction efficiency was assessed using a droplet digital PCR (ddPCR) copy number variation assay using gDNA of the heart and other organs with high AAV9 tropism (Fig. 5c-g). The transduction efficiency was calculated by quantifying bGHpA (Fig. 5c) and Magel2 sequences (Fig. 5d) to determine AAV viral genome and host genome copies, respectively. The analysis of normalized viral genome copies indicated that AAV application via the retro-orbital sinus yielded a 2-fold higher transduction efficiency (1.2 AAV copies/host genome) in the myocardium, compared to tail vein injections in which 0.6 AAV copies/host genome were detected (Fig. 5e). In addition, the viral load in the liver, the organ with the highest AAV9 tropism, was significantly reduced by retro-orbital application (7.8 AAV copies/host genome) compared to tail vein injections (12.4 AAV copies/host genome, Fig. 5f), while the route of application showed no significant difference in skeletal muscle transduction (Fig. 5 g).

We then injected juvenile wild-type mice showing high cardiac transduction efficiency with the scAAV9-shTRPM4^miR30^-tandem construct via the retro-orbital sinus. As an AAV9 transduction control, siblings were treated with a scAAV9-eGFP in the same way (Fig. 5h). As before, the mice were analyzed 16 weeks after AAV9 application (Fig. 5i). Hearts were subjected to microsomal membrane purification and TRPM4 protein expression was analyzed by Western blotting (Fig. 5j). Densitometric analysis showed a significant reduction in TRPM4 protein levels by 90 ± 7% in mice treated with scAAV9-CMV-shTRPM4^mir30^-tandem compared to siblings treated with the scAAV9-eGFP control (Fig. 5k).

## Discussion

Heart failure represents one of today’s largest global health problems and improved therapy options are urgently required. In light of the recent clinical success of several gene therapies for monogenic diseases, cancer gene therapy is primed to be a most promising future option for the treatment of heart failure [7, 33, 42]. Target molecules for this include regulators of myocardial Ca^2+^ signaling, which is often derailed in the failing heart. Several previous studies have indicated that activation of the TRPM4 cation channel in cardiomyocytes negatively modulates the L-type Ca_v_ channel-mediated Ca^2+^ influx. Global knockout of TRPM4 in mouse led to increased cardiac contractility under β-adrenergic stimulation, both under basal conditions and after induced myocardial infarction [15, 23, 37]. This raised the hypothesis that inhibition of TRPM4 in the heart could lead to an increase in cardiac contractility in patients with elevated catecholamine levels. So far, no specific low molecular weight TRPM4 blockers are available. Since TRPM4 is expressed not only in cardiomyocytes but also in many other cells, the use of such blockers might lead to severe adverse drug effects, such as increasing arterial blood pressure [22], triggering an anaphylactic reaction through the release of pro-inflammatory factors from mast cells [38], and increasing activation of immune cells such as dendritic cells [2] or T cells [19, 40]. Therefore, gene transfer of an RNAi sequence using recombinant AAV9 vectors to specifically target the TRPM4 transcript in cardiomyocytes may be a safer strategy. Cardiomyocytes can be transduced with high efficiency using AAV9 vectors and, with the help of cardiomyocyte-derived promoters in the corresponding vectors, the expression and the associated knockdown of TRPM4 can be confined to the contractile myocardium with only limited expression in other organs even when AAV particles are applied systemically [27, 41].

In this work, specific shRNA^miR30^ sequences against the mRNA of the murine TRPM4 and distinct vector designs were evaluated. These included a microRNA-based RNAi design, which has an advantageous safety profile with respect to oversaturation of miRNA processing pathway [5, 9] and can also be expressed by tissue-specific promoters as a multi-shRNA^miR30^ transcript [39].

At the beginning of our study, various shRNA^miR30^ sequences were screened in vitro for their efficiency in degrading the murine Trpm4 mRNA and in thus causing TRPM4 knockdown. We used the murine cell line B16F10, because they were found to exhibit sufficiently high endogenous expression of TRPM4 proteins for our screening of knockout approach with shRNA^miR30^ constructs. Compared to cardiomyocytes or heart tissue, the B16F10 cells are available in unlimited amounts or can be expanded indefinitely. This is important because for a reliable quantitative determination of TRPM4 protein levels using Western blotting, membrane protein fractions of B16F10 cells must first be prepared. In order to avoid a dilution of the knockdown effect after several rounds of cell division, as would have been the case with transient transfection approaches as well with an AAV6-mediated gene transfer in which the transgene is not transmitted to daughter cells, the shRNA^miR30^ constructs were packaged as lentiviruses. The benefit is that transduction with lentiviral vectors leads to stable integration of the transgene cassette into the host genome. The lentiviral approach has the additional advantage that daughter cells also carry the transgenic information and that the transduced cells can be expanded. In addition, the transgenic cassette contained a puromycin resistance gene, which allowed for a depletion of non-transduced cells before expansion. As a result, despite a low initial transduction efficiency, cells that expressed the shRNA^miR30^ construct could be specifically expanded and analyzed, which alleviated the expression analysis of low abundant TRPM4 proteins. Using the lentiviral-based in vitro screen, we identified three shRNA^miR30^ sequences from a total of six, which reduced the endogenous TRPM4 protein level by up to 90 ± 6%.

The primary goal of this work was to downregulate the TRPM4 protein level in the adult murine heart to a maximum extent that is comparable to complete gene deletion as obtained by Cre-loxP-dependent TRPM4 deletion in cardiomyocytes. The two most potent shRNA^miR30^ sequences from the in vitro screen were therefore selected for an in vivo TRPM4 knockdown strategy suitable for therapeutic approaches and subcloned as a tandem construct under the control of the cardiomyocyte-specific MLC260 promoter. For in vivo gene transfer, we chose AAV9 vectors at a dose of 3 × 10^12^ viral genomes per mouse and initially tested application via intravenous administration, since this strategy has already been described to achieve homogeneous and effective transduction efficiency in the left ventricle [3, 14, 21, 34, 41, 43].

In a first series of experiments, an ssAAV genome was used, which contained the shRNA^miR30^-tandem construct. TRPM4 knockdown efficiency was less than 50%, 11 weeks after AAV9 application. Since this degree of reduction is probably too low to expect an increased cardiac contractility, various features of the AAV-shRNA construct were further optimized. We aimed to enhance knockdown efficiency by the use of scAAV vectors which can achieve a faster and more efficient expression of the transgenic cassette [25]. Because scAAVs allow only reduced packaging capacity, we had to remove the expression reporter (tdTomato) as well as the WPRE sequence so that the transgene cassette was reduced to the promoter, the shRNA^miR^-tandem, and the polyadenylation sequence.

Furthermore, an increase in the expression level of the shRNA^miR30^-tandem should be achieved by using the CMV RNA Pol II promoter. Compared to promoters derived from cardiomyocytes, the CMV promoter mediates a significantly higher expression level in the mouse myocardium [6, 27, 34]. However, the degree of TRPM4 downregulation using this shRNA^miR30^-tandem construct in the scAAV backbone under the control of the CMV promotor was also only in the range of 50% after 11 weeks, and slightly improved (~ 60%) when evaluated 16 weeks after vector application, supporting the idea that extending the life-span period is beneficial. In addition to the shRNAmiR30 RNAi approach, a classical shRNA design was developed as an alternative RNAi approach and tested directly in vivo without prior in vitro evaluation. However, the analysis of the shRNA-triple construct did not increase the TRPM4 knockdown efficiency compared to the previous ssAAV-shRNA^miR30^-tandem vector. Due to concerns about shRNA toxicity and the restriction to expression from RNA Pol III promoters, no further test groups were treated with the classic shRNA-triple vector in order to increase the TRPM4 knockdown.

Since the knockdown potential of shRNA-#4 was in the range of 90% in B16F10 cells, we reasoned that the lower knockdown efficiency in the heart observed so far might result from inefficient delivery to the heart and cardiomyocytes by application to the peripheral circulation via the tail vein. We therefore tested whether injection via the retro-orbital sinus may lead to improved transduction efficiency with a given AAV9 vector. In a first experiment, we thus compared head to head the application via the retro-orbital sinus with injections via the lateral tail vein and found 2-fold more AAV viral copies per host genome with the application route via the retro-orbital sinus. This result strengthens the hypotheses that the injection via the retro-orbital sinus improves cardiac AAV9 transduction by allowing the vector solution to reach the heart more directly without significant dilution in the circulation. The reduced transduction efficiency in the liver observed with injection via the retro-orbital route is an additional argument for this hypothesis. Since the retro-orbital injection can already be carried out accurately in 3-week-old mice, juvenile mice were used in order to minimize the distribution volume of the vector solution as much as possible although it has been reported that the AAV vector transduction is more stable in adult animals [12]. Indeed, a ~90% reduction in TRPM4 protein expression was achieved with the latter approach.

Given that no more than 90% reduction in the ventricle was obtained by Cre-loxP-mediated gene deletion in a cardiomyocyte-specific knockout mouse, it can be assumed that the improved AAV9-shTRPM4^miR30^-tandem-induced knockdown of TRPM4 in cardiomyocytes should be close to 100%. This result suggests that the retro-orbital application route in combination with the small distribution volume of juvenile mice achieves an improved AAV9 transfer into the heart of the animals and consequently leads to an increased cardiac TRPM4 knockdown. This gene silencing strategy can now be used to treat preclinical mouse models of acute and chronic systolic heart failure in order to restore cardiac contractile function.

Taken together, we have successfully developed a highly efficient AAV9-mediated RNAi strategy to achieve a vector-induced knockdown of TRPM4 in the adult mouse heart. Combining the robust AAV9-shRNA^miR30^ design with an optimal route of application yielded a 90% reduction of TRPM4 in the mouse myocardium. This strategy represents a promising approach to test whether TRPM4 knockdown leads to an increase in inotropic response in healthy animals and particularly in heart failure models. If successful, the strategy could be transferred to patients with heart failure associated with elevated catecholamine levels.

## Supplementary information

Fig. S1 (DOCX 144 kb)

Fig. S2 (DOCX 110 kb)

Fig. S3 (DOCX 635 kb)

Table S1. (DOCX 21 kb)

Table S2. (DOCX 21 kb)

## Data Availability

All data that support the findings of this study are available in this study within the manuscript and/or its supplementary materials.

## References

[1] Adams D, Gonzalez-Duarte A, O’Riordan WD, Yang CC, Ueda M, Kristen AV, Tournev I, Schmidt HH, Coelho T, Berk JL, Lin KP, Vita G, Attarian S, Plante-Bordeneuve V, Mezei MM, Campistol JM, Buades J, Brannagan TH, Kim BJ, Oh J, Parman Y, Sekijima Y, Hawkins PN, Solomon SD, Polydefkis M, Dyck PJ, Gandhi PJ, Goyal S, Chen J, Strahs AL, Nochur SV, Sweetser MT, Garg PP, Vaishnaw AK, Gollob JA, Suhr OB (2018). Patisiran, an RNAi therapeutic, for hereditary transthyretin amyloidosis. N Engl J Med.

[2] Barbet G, Demion M, Moura IC, Serafini N, Leger T, Vrtovsnik F, Monteiro RC, Guinamard R, Kinet JP, Launay P (2008). The calcium-activated nonselective cation channel TRPM4 is essential for the migration but not the maturation of dendritic cells. Nat Immunol.

[3] Bish LT, Morine K, Sleeper MM, Sanmiguel J, Wu D, Gao G, Wilson JM, Sweeney HL (2008). Adeno-associated virus (AAV) serotype 9 provides global cardiac gene transfer superior to AAV1, AAV6, AAV7, and AAV8 in the mouse and rat. Hum Gene Ther.

[4] Borner K, Kienle E, Huang LY, Weinmann J, Sacher A, Bayer P, Stullein C, Fakhiri J, Zimmermann L, Westhaus A, Beneke J, Beil N, Wiedtke E, Schmelas C, Miltner D, Rau A, Erfle H, Krausslich HG, Muller M, Agbandje-McKenna M, Grimm D (2020). Pre-arrayed Pan-AAV peptide display libraries for rapid single-round screening. Mol Ther.

[5] Boudreau RL, Martins I, Davidson BL (2009). Artificial microRNAs as siRNA shuttles: improved safety as compared to shRNAs in vitro and in vivo. Mol Ther.

[6] Geisler A, Jungmann A, Kurreck J, Poller W, Katus HA, Vetter R, Fechner H, Muller OJ (2011). microRNA122-regulated transgene expression increases specificity of cardiac gene transfer upon intravenous delivery of AAV9 vectors. Gene Ther.

[7] Greenberg B (2017). Gene therapy for heart failure. Trends Cardiovasc Med.

[8] Grieger JC, Choi VW, Samulski RJ (2006). Production and characterization of adeno-associated viral vectors. Nat Protoc.

[9] Grimm D (2011). The dose can make the poison: lessons learned from adverse in vivo toxicities caused by RNAi overexpression. Silence.

[10] Grimm D, Kay MA (2007). Therapeutic application of RNAi: is mRNA targeting finally ready for prime time?. J Clin Invest.

[11] Grimm D, Streetz KL, Jopling CL, Storm TA, Pandey K, Davis CR, Marion P, Salazar F, Kay MA (2006). Fatality in mice due to oversaturation of cellular microRNA/short hairpin RNA pathways. Nature.

[12] Han SO, Li S, McCall A, Arnson B, Everitt JI, Zhang H, Young SP, ElMallah MK, Koeberl DD (2020). Comparisons of infant and adult mice reveal age effects for liver depot gene therapy in Pompe disease. Mol Ther Methods Clin Dev.

[13] Hovingh GK, Lepor NE, Kallend D, Stoekenbroek RM, Wijngaard PLJ, Raal FJ (2020). Inclisiran durably lowers low-density lipoprotein cholesterol and proprotein convertase subtilisin/kexin type 9 expression in homozygous familial hypercholesterolemia: the ORION-2 Pilot Study. Circulation.

[14] Inagaki K, Fuess S, Storm TA, Gibson GA, McTiernan CF, Kay MA, Nakai H (2006). Robust systemic transduction with AAV9 vectors in mice: efficient global cardiac gene transfer superior to that of AAV8. Mol Ther.

[15] Jacobs G, Oosterlinck W, Dresselaers T, Geenens R, Kerselaers S, Himmelreich U, Herijgers P, Vennekens R (2015). Enhanced beta-adrenergic cardiac reserve in Trpm4(-)/(-) mice with ischaemic heart failure. Cardiovasc Res.

[16] Jungmann A, Leuchs B, Katus HA, Rommelaere J, Muller OJ (2017) Protocol for efficient generation and characterization of adeno-associated viral (AAV) vectors. Hum Gene Ther Methods. 10.1089/hum.2017.192 10.1089/hgtb.2017.192 29048971

[17] Kruse M, Schulze-Bahr E, Corfield V, Beckmann A, Stallmeyer B, Kurtbay G, Ohmert I, Schulze-Bahr E, Brink P, Pongs O (2009). Impaired endocytosis of the ion channel TRPM4 is associated with human progressive familial heart block type I. J Clin Invest.

[18] Launay P, Fleig A, Perraud AL, Scharenberg AM, Penner R, Kinet JP (2002). TRPM4 is a Ca2+-activated nonselective cation channel mediating cell membrane depolarization. Cell.

[19] Launay P, Cheng H, Srivatsan S, Penner R, Fleig A, Kinet JP (2004). TRPM4 regulates calcium oscillations after T cell activation. Science.

[20] Liu H, El Zein L, Kruse M, Guinamard R, Beckmann A, Bozio A, Kurtbay G, Megarbane A, Ohmert I, Blaysat G, Villain E, Pongs O, Bouvagnet P (2010). Gain-of-function mutations in TRPM4 cause autosomal dominant isolated cardiac conduction disease. Circ Cardiovasc Genet.

[21] Long C, Amoasii L, Mireault AA, McAnally JR, Li H, Sanchez-Ortiz E, Bhattacharyya S, Shelton JM, Bassel-Duby R, Olson EN (2016). Postnatal genome editing partially restores dystrophin expression in a mouse model of muscular dystrophy. Science.

[22] Mathar I, Vennekens R, Meissner M, Kees F, Van der Mieren G, Camacho Londono JE, Uhl S, Voets T, Hummel B, van den Bergh A, Herijgers P, Nilius B, Flockerzi V, Schweda F, Freichel M (2010). Increased catecholamine secretion contributes to hypertension in TRPM4-deficient mice. J Clin Invest.

[23] Mathar I, Kecskes M, Van der Mieren G, Jacobs G, Camacho Londono JE, Uhl S, Flockerzi V, Voets T, Freichel M, Nilius B, Herijgers P, Vennekens R (2014). Increased beta-adrenergic inotropy in ventricular myocardium from Trpm4-/- mice. Circ Res.

[24] McBride JL, Boudreau RL, Harper SQ, Staber PD, Monteys AM, Martins I, Gilmore BL, Burstein H, Peluso RW, Polisky B, Carter BJ, Davidson BL (2008). Artificial miRNAs mitigate shRNA-mediated toxicity in the brain: implications for the therapeutic development of RNAi. Proc Natl Acad Sci U S A.

[25] McCarty DM, Monahan PE, Samulski RJ (2001). Self-complementary recombinant adeno-associated virus (scAAV) vectors promote efficient transduction independently of DNA synthesis. Gene Ther.

[26] Medert R, Pironet A, Bacmeister L, Segin S, Londono JEC, Vennekens R, Freichel M (2020). Genetic background influences expression and function of the cation channel TRPM4 in the mouse heart. Basic Res Cardiol.

[27] Muller OJ, Leuchs B, Pleger ST, Grimm D, Franz WM, Katus HA, Kleinschmidt JA (2006). Improved cardiac gene transfer by transcriptional and transductional targeting of adeno-associated viral vectors. Cardiovasc Res.

[28] Nakagaki A, Urakawa A, Hirano S, Anami T, Kishino T (2018). Application of droplet digital PCR in the analysis of genome integration and organization of the transgene in BAC transgenic mice. Sci Rep.

[29] Nelson PL, Zolochevska O, Figueiredo ML, Soliman A, Hsu WH, Feng JM, Zhang H, Cheng H (2011). Regulation of Ca(2+)-entry in pancreatic alpha-cell line by transient receptor potential melastatin 4 plays a vital role in glucagon release. Mol Cell Endocrinol.

[30] Nelson P, Ngoc Tran TD, Zhang H, Zolochevska O, Figueiredo M, Feng JM, Gutierrez DL, Xiao R, Yao S, Penn A, Yang LJ, Cheng H (2013). Transient receptor potential melastatin 4 channel controls calcium signals and dental follicle stem cell differentiation. Stem Cells.

[31] Pacak CA, Mah CS, Thattaliyath BD, Conlon TJ, Lewis MA, Cloutier DE, Zolotukhin I, Tarantal AF, Byrne BJ (2006). Recombinant adeno-associated virus serotype 9 leads to preferential cardiac transduction in vivo. Circ Res.

[32] Pacak CA, Sakai Y, Thattaliyath BD, Mah CS, Byrne BJ (2008). Tissue specific promoters improve specificity of AAV9 mediated transgene expression following intra-vascular gene delivery in neonatal mice. Genet Vacc Ther.

[33] Pleger ST, Brinks H, Ritterhoff J, Raake P, Koch WJ, Katus HA, Most P (2013). Heart failure gene therapy: the path to clinical practice. Circ Res.

[34] Prasad KM, Xu Y, Yang Z, Acton ST, French BA (2011). Robust cardiomyocyte-specific gene expression following systemic injection of AAV: in vivo gene delivery follows a Poisson distribution. Gene Ther.

[35] Stallmeyer B, Zumhagen S, Denjoy I, Duthoit G, Hebert JL, Ferrer X, Maugenre S, Schmitz W, Kirchhefer U, Schulze-Bahr E, Guicheney P, Schulze-Bahr E (2012). Mutational spectrum in the Ca(2+)-activated cation channel gene TRPM4 in patients with cardiac conductance disturbances. Hum Mutat.

[36] Takefuji M, Wirth A, Lukasova M, Takefuji S, Boettger T, Braun T, Althoff T, Offermanns S, Wettschureck N (2012). G(13)-mediated signaling pathway is required for pressure overload-induced cardiac remodeling and heart failure. Circulation.

[37] Uhl S, Mathar I, Vennekens R, Freichel M (2014). Adenylyl cyclase-mediated effects contribute to increased Isoprenaline-induced cardiac contractility in TRPM4-deficient mice. J Mol Cell Cardiol.

[38] Vennekens R, Olausson J, Meissner M, Bloch W, Mathar I, Philipp SE, Schmitz F, Weissgerber P, Nilius B, Flockerzi V, Freichel M (2007). Increased IgE-dependent mast cell activation and anaphylactic responses in mice lacking the calcium-activated nonselective cation channel TRPM4. Nat Immunol.

[39] Wang J, Theunissen TW, Orkin SH (2007). Site-directed, virus-free, and inducible RNAi in embryonic stem cells. Proc Natl Acad Sci U S A.

[40] Weber KS, Hildner K, Murphy KM, Allen PM (2010). Trpm4 differentially regulates Th1 and Th2 function by altering calcium signaling and NFAT localization. J Immunol.

[41] Werfel S, Jungmann A, Lehmann L, Ksienzyk J, Bekeredjian R, Kaya Z, Leuchs B, Nordheim A, Backs J, Engelhardt S, Katus HA, Muller OJ (2014). Rapid and highly efficient inducible cardiac gene knockout in adult mice using AAV-mediated expression of Cre recombinase. Cardiovasc Res.

[42] Zhang Y, Li H, Min YL, Sanchez-Ortiz E, Huang J, Mireault AA, Shelton JM, Kim J, Mammen PPA, Bassel-Duby R, Olson EN (2020). Enhanced CRISPR-Cas9 correction of Duchenne muscular dystrophy in mice by a self-complementary AAV delivery system. Sci Adv.

[43] Zincarelli C, Soltys S, Rengo G, Koch WJ, Rabinowitz JE (2010). Comparative cardiac gene delivery of adeno-associated virus serotypes 1-9 reveals that AAV6 mediates the most efficient transduction in mouse heart. Clin Transl Sci.

[44] Zolotukhin S, Byrne BJ, Mason E, Zolotukhin I, Potter M, Chesnut K, Summerford C, Samulski RJ, Muzyczka N (1999). Recombinant adeno-associated virus purification using novel methods improves infectious titer and yield. Gene Ther.

