# Supplementary material for: Development of an AAV9-RNAi-mediated silencing strategy to abrogate TRPM4 expression in the adult heart
Source: Pflugers Arch. 2021 Feb 13;473(3):533–46. doi: 10.1007/s00424-021-02521-6 (PMC7940300; doi:10.1007/s00424-021-02521-6)
Supplement: Supplementary file 1 — (DOCX 144 kb) [file 424_2021_2521_MOESM1_ESM.docx]

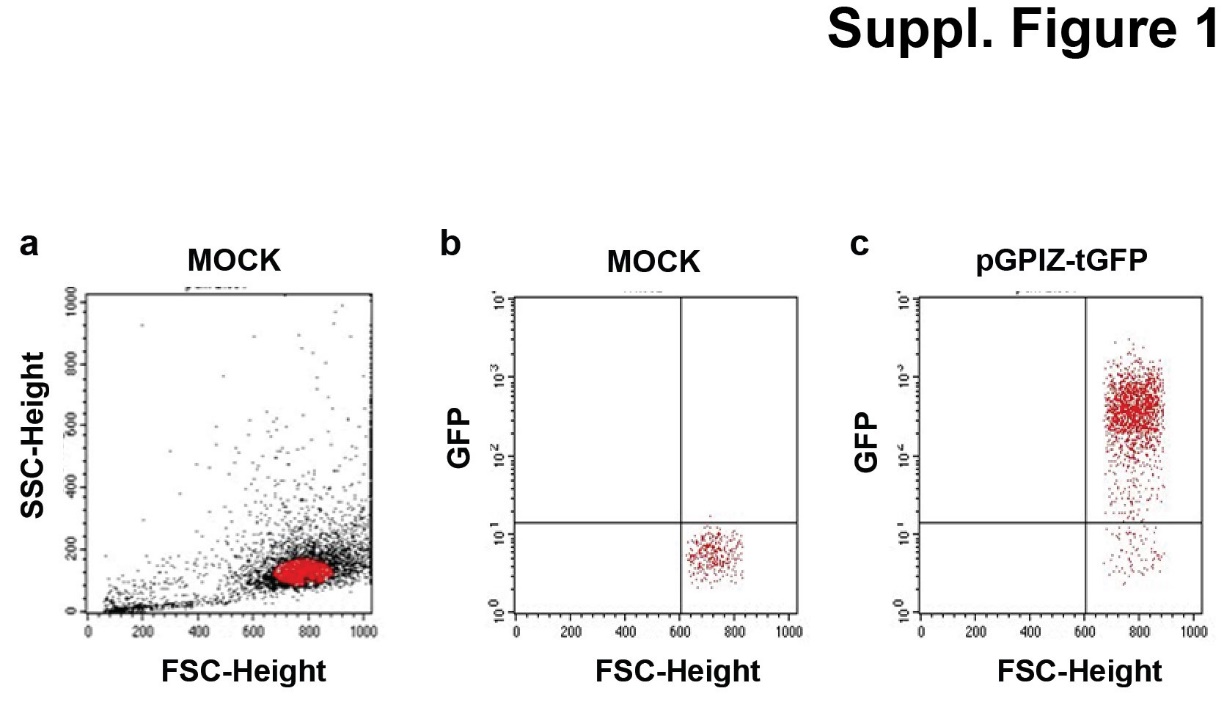


**Fig. S1** Lentiviral transduction of shTRPM4^miR30^ constructs in B16F10 cells. FACS analysis of GFP fluorescence signal in puromycin-selected B16F10 cells 13 days after lentiviral transduction of shTRPM4^miR30^ constructs.
