# Supplementary material for: Development of an AAV9-RNAi-mediated silencing strategy to abrogate TRPM4 expression in the adult heart
Source: Pflugers Arch. 2021 Feb 13;473(3):533–46. doi: 10.1007/s00424-021-02521-6 (PMC7940300; doi:10.1007/s00424-021-02521-6)
Supplement: Supplementary file 2 — (DOCX 110 kb) [file 424_2021_2521_MOESM2_ESM.docx]

**
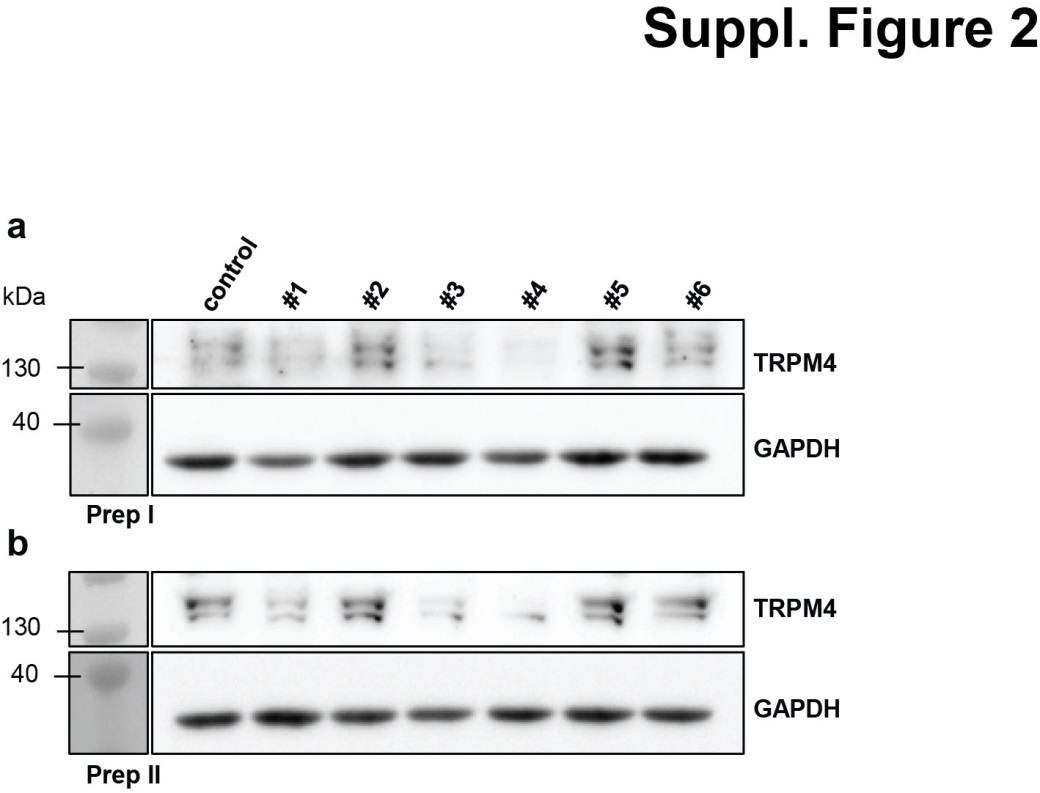
**

**Fig. S2** Western blot analysis of shTRPM4^miR30^(# 1- # 6) mediated TRPM4 knockdown in microsomal membrane fractions of B16F10 cells. Shown are two technical replicates of the Western blot quantification in Fig. 2.
