# Supplementary material for: Development of an AAV9-RNAi-mediated silencing strategy to abrogate TRPM4 expression in the adult heart
Source: Pflugers Arch. 2021 Feb 13;473(3):533–46. doi: 10.1007/s00424-021-02521-6 (PMC7940300; doi:10.1007/s00424-021-02521-6)
Supplement: Supplementary file 3 — (DOCX 635 kb) [file 424_2021_2521_MOESM3_ESM.docx]

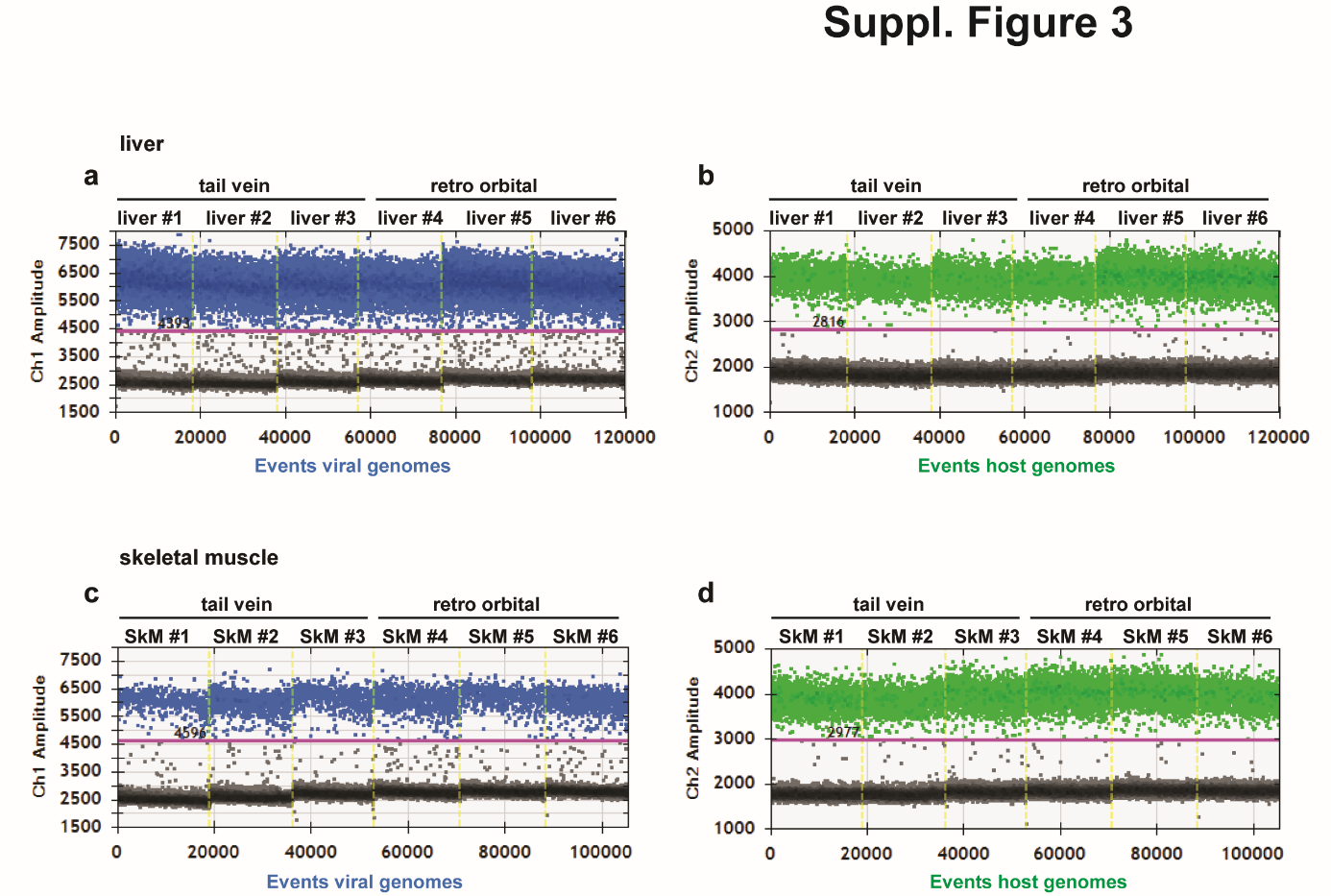


**Fig. S3** ddPCR droplet count view for **(c)** viral genomes and **(d)** host genomes in liver and skeletal muscle (SkM, Musculus vastus lateralis) injected via tail vein or retro-orbital sinus. Positive droplets are displayed in blue (viral genome counts of the AAV containing bGHpA, bovine growth hormone polyadenylation signal sequence) or green (host genome counts of the Magel2 reference gene) and negative droplets in black. Quantification is displayed in Fig 5.
