# Supplementary material for: Development of an AAV9-RNAi-mediated silencing strategy to abrogate TRPM4 expression in the adult heart
Source: Pflugers Arch. 2021 Feb 13;473(3):533–46. doi: 10.1007/s00424-021-02521-6 (PMC7940300; doi:10.1007/s00424-021-02521-6)
Supplement: Supplementary file 4 — (DOCX 21 kb) [file 424_2021_2521_MOESM4_ESM.docx]

| **Name** | **Target DNA sequence** |
| --- | --- |
| **shTRPM4^miR-30^ (#1)** | 5´ CCTAACTCACTGATCCGAAAT 3´ |
|  | 3´ GGATTGAGTGACTAGGCTTTA 5´ |
| **shTRPM4^miR-30^ (#2)** | 5´ GCGAAGCCCGGGATCGGATTA 3´ |
|  | 3´ CGCTTCGGGCCCTAGCCTAAT 5´ |
| **shTRPM4^miR-30^ (#3)** | 5´ TTGGAGAGTGTTACCACAACA 3´ |
|  | 3´ AACCTCTCACAATGGTGTTGT 5´ |
| **shTRPM4^miR-30^ (#4)** | 5´ AAAGGAGTTGCTGACGGTCTA 3´ |
|  | 3´ TTTCCTCAACGACTGCCAGAT 5´ |
| **shTRPM4^miR-30^ (#5)** | 5´ CGCACATCTTCACGGTGAACAA 3´ |
|  | 3´ GCGTGTAGAAGTGCCACTTGTT 5´ |
| **shTRPM4^miR-30^ (#6)** | 5´ CGAGAAGATGTTAAAGCGGATA 3´ |
|  | 3´ GCTCTTCTACAATTTCGCCTAT 5´ |
| **shTRPM4 (#4)** | 5´ AAAGGAGTTGCTGACGGTCTA 3´ |
|  | 3´ TTTCCTCAACGACTGCCAGAT 5´ |
| **shTRPM4(#7)** | 5´ GAAGATGTTAAAGCGGATAGA 3´ |
|  | 3´ CTTCTACAATTTCGCCTATCT 5´ |
| **shTRPM4(#8)** | 5´ GGCCCAAGATTGTCATAGTGA 3´ |
|  | 3´ CCGGGTTCTAACAGTATCACT 5´ |

**Table S1.** DNA sequences of the shTRPM4miR30 and shRNA target DNA sequences used.
