# Supplementary material for: Development of an AAV9-RNAi-mediated silencing strategy to abrogate TRPM4 expression in the adult heart
Source: Pflugers Arch. 2021 Feb 13;473(3):533–46. doi: 10.1007/s00424-021-02521-6 (PMC7940300; doi:10.1007/s00424-021-02521-6)
Supplement: Supplementary file 5 — (DOCX 21 kb) [file 424_2021_2521_MOESM5_ESM.docx]

| **Name** | **Oligo DNA sequence or assay ID** |
| --- | --- |
| **Trpm4 WT/Flox-fwd** | 5´ ACCTACAGGAAACCTCGGGG 3´ |
| **Trpm4 WT/Flox-rev** | 5´ GTTTGATGTCTCCTTCAGTCG 3´ |
| **Trpm4 KO-fwd** | 5´ GAGTTCCTGTCCTCCTAAAGG 3´ |
| **Trpm4 KO-rev** | 5´ GTTTGATGTCTCCTTCAGTCG 3´ |
| **αMHC/CreERT2-fwd** | 5´ TTATGGTACCACATAGACCTCT 3´ |
| **αMHC/CreERT2-rev** | 5´ TGCTGTTGGATGGTCTTCACAG 3´ |
| **bGHpA-fwd_1** | 5´ ACCTAACTCACTGATCCGAAATTA 3´ |
| **bGHpA-rev_1** | 5´ ATTTCGGATCAGTGAGTTAGG 3´ |
| **bGHpA-fwd_2** | 5´ ACCTAACTCACTGATCCGAAATTA 3´ |
| **bGHpA-rev_2** | 5´ ATTTCGGATCAGTGAGTTAGG 3´ |
| **Magel2-fwd** | BioRad assay ID: dMmuCNS345882175 |
| **Magel2-rev** | BioRad assay ID: dMmuCNS345882175 |
| **bGHpA probe** | 5´ 6FAM-TCCCCCGTGCCTTCCTTGACC-BHQ1 3´ |
| **Magel2 probe** | BioRad assay ID: dMmuCNS345882175 |

**Table S2.** Overview of primer sets and probes used for genotyping, qPCR and ddPCR.
